# A phase II study of poziotinib in patients with recurrent and/or metastatic head and neck squamous cell carcinoma

**DOI:** 10.1002/cam4.4231

**Published:** 2021-09-16

**Authors:** Ji Hyun Lee, Seong Gu Heo, Beung‐Chul Ahn, Min Hee Hong, Byoung Chul Cho, Sun Min Lim, Hye Ryun Kim

**Affiliations:** ^1^ Division of Medical Oncology Department of Internal Medicine Yonsei University College of Medicine Yonsei Cancer Centre Seoul Republic of Korea; ^2^ Severance Biomedical Science Institute Yonsei University College of Medicine Seoul Republic of Korea

**Keywords:** biomarker, head and neck cancer, poziotinib

## Abstract

**Background:**

In phase I studies, poziotinib has shown meaningful efficacy against various types of cancers. This phase 2 study aimed to investigate the efficacy and safety of poziotinib in recurrent and/or metastatic head and neck squamous cell carcinoma (R/M‐HNSCC).

**Methods:**

Overall, 49 patients were enrolled (median age, 62 years; age range, 21–78 years). Patients received a median of two prior treatments including chemotherapy and others and received 12 mg poziotinib orally once daily as part of a 28‐day cycle. The primary endpoint was objective response rate (ORR), and the secondary endpoints were progression‐free survival (PFS) and overall survival (OS). Targeted capture sequencing was performed using available tissues to identify translational biomarkers related to clinical response.

**Results:**

ORR was 22.4%, median PFS was 4.0 months (95% confidence interval [CI], 1.8–6.2 months), and median OS was 7.6 months (95% CI, 4.4–10.8 months). The most common treatment‐related adverse events were acneiform rash (85%) and mucositis (77%). A grade 3 or higher adverse event was acneiform rash (3%). Targeted capture sequencing was performed in 30 tissue samples. *TP53* and *PIK3CA* were the most frequently mutated genes (43%), followed by *CCND1* (33%) and *EGFR* (30%). Mutations in *ERBB2*, *ERBB3*, and *ERBB4*, which are HER family genes, were observed in 17%, 13%, and 10% samples, respectively. There was no difference in the frequency of somatic mutations in the HER family genes between the clinically benefitted and non‐benefitted groups.

**Conclusion:**

Compared to other pan‐HER inhibitors, poziotinib showed clinically meaningful efficacy in heavily treated R/M‐HNSCC.

**Clinical trial registration number.:**

NCT02216916.

## Graphical abstract

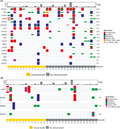

## 1 INTRODUCTION

Head and neck squamous cell carcinoma (HNSCC) is the sixth most common cancer worldwide.^1^, ^2^ Even in advanced disease, cure is possible through multi‐modality therapy. However, recurrent and/or metastatic HNSCC (R/M‐HNSCC) has poor prognosis, with a median survival of 6–10 months^3^, ^4^ and 50–60% patients develop loco‐regional or distant recurrence within 2 years.

Epithelial growth factor receptor (EGFR) is frequently overexpressed in HNSCC and is associated with poor prognosis.^5^ The relationship between the EGFR signaling pathway and tumor survival is well known, as demonstrated in several studies. EGFR targeted therapies, especially cetuximab, show clinical anticancer effects in HNSCC.^3^ In the EXTREME study, when 5FU and cetuximab were added to platinum chemotherapy as the primary drug, the median OS was 10.1 months compared with 7.4 months when only 5FU and platinum chemotherapy were used.^6^ However, cetuximab is the only approved targeted agent for HNSCC, with a response rate of 10–15% in patients with R/M‐HNSCC.^7^

Anti‐programmed cell death 1 (PD‐1) agents, including pembrolizumab and nivolumab, were recently approved for HNSCC that is refractory to platinum‐based therapy.^8^, ^9^, ^10^ In the Keynote‐048 trial, pembrolizumab and pembrolizumab +platinum drug +5‐FU were used as a new first‐line standard of treatment for R/M‐HNSCC.^11^ However, the objective response was modest, and more effective treatment strategies are needed. In addition to immunotherapy, novel targeted therapies, such as tyrosine kinase inhibitor (TKI), should be developed.

Genomic characterization of HNSCC has recently been reported, and amplification of receptor tyrosine kinases, including *EGFR* and *ERBB2*, was commonly identified in HPV‐negative HNSCC.^12^ The amplification of *EGFR* was reported in 15% cases and that of ERBB2 was reported in 5%, making these the most common gene amplifications. Therefore, *EGFR* and *ERBB2* remain viable therapeutic targets for patients with HNSCC.^12^

Poziotinib (HM781‐36B) is an irreversible pan‐HER TKI that targets EGFR, HER2, and HER4.^13^ It binds to the HER family of tyrosine kinase receptors and blocks downstream signaling pathway. Therefore, given the public genomic data that EGFR and ERBB2 play an important role in the carcinogenesis of HNSCC, we attempted to administer poziotinib to patients to examine its efficacy and tolerability. In phase I clinical trials, poziotinib showed notable clinical activity against various types of solid tumors.^13^, ^14^

In this study, we aimed to demonstrate the efficacy and safety of poziotinib in heavily treated R/M‐HNSCC and identify translational biomarkers related to clinical response to poziotinib.

## 2 MATERIALS AND METHODS

### 2.1 Study design

This study was a single‐center, phase II trial of poziotinib monotherapy in R/M‐HNSCC patients who exhibited disease progression after platinum‐based chemotherapy or were not eligible for platinum‐based chemotherapy (ClinicalTrials.gov Identifier: NCT02216916). The objective response rate (ORR) was the primary endpoint, whereas progression‐free survival (PFS), OS, and the safety profile of poziotinib therapy were the secondary endpoints.

This study was approved by the Institutional Review Board of Severance Hospital (4–2013–0794). The study conforms to the principles for research outlined in the Declaration of Helsinki. Written informed consent was obtained from all patients before study enrolment.

### 2.2 Study population

The subjects were histologically confirmed R/M‐HNSCC patients in Yonsei Cancer Center. Patients were eligible for enrolment if they were aged above 20 years, with an Eastern Cooperative Oncology Group performance status (ECOG‐PS) of 0 to 1, with at least one measurable disease based on Response Evaluation Criteria In Solid Tumors (RECIST, version 1.1), with documented progression after platinum‐based systemic chemotherapy, and with a life expectancy of at least 3 months were included. Chemotherapy‐naive patients who had inadequate renal function for platinum administration could be enrolled in the study.

Patients with more than three lines of previous palliative chemotherapy for R/M‐ HNSCC and previous EGFR tyrosine kinase treatments were excluded, except for those who had received cetuximab. Patients with nasopharyngeal cancer or symptomatic brain metastases were also excluded.

### 2.3 Treatment plan

Patients were continuously treated with 12 mg oral poziotinib once daily until disease progression, death, or unacceptable adverse events (AEs). The duration of the treatment cycle was 28 days. Drug doses were held and/or reduced for intolerable grade 2 or 3/4 adverse events. Up to two dose reductions was allowed (8 mg followed by 6 mg). If treatment was not resumed within 3 weeks, patients discontinued the study.

### 2.4 Assessment

Response evaluation was performed every 8 weeks after disease progression or when clinically indicated according to RECIST 1.1 guidelines.^15^

Safety assessments included physical examination, evaluation of AEs, and laboratory tests on day 1 of each cycle. All AEs were documented according to the Common Terminology Criteria for Adverse Events version 4.03.^16^ Patients with clinical benefits were defined as those with PFS ≥6 months using poziotinib.

### 2.5 Somatic mutation and copy number alteration analysis

Formalin‐fixed, paraffin‐embedded tumor tissues, and blood samples were prepared using the Agilent SureSelect Target Enrichment Kit (Agilent Technologies, Inc.). Targeted deep sequencing data were generated using Illumina NovaSeq 6000 (Illumina) with a read length of 101 bp and a mean depth of target regions of 1,000X. Read alignment and somatic mutation calling were performed using the DNA Pipeline of the Illumina DRAGEN Bio‐IT Platform v3.6 (Illumina). Tumor somatic mutation annotation was performed using Oncotator v1.9.9.0. Somatic copy number alterations (SCNA) were called using CNVkit v0.9.5. A combined somatic mutation and SCNA oncoplot was drawn using ComplexHeatmap v2.4.3. Copy number alterations were defined as copy number amplifications (copy number > 5) and copy number deletions (copy number = 1 or 0).

### 2.6 Statistical rationale for the study design

Statistical design was carried out according to Fleming's one‐stage design. The null hypothesis (P0) with a 5% significance level that the ORR is ≤5% versus the alternative hypothesis (P1) that the ORR is ≥15% was evaluated. Forty‐four response‐evaluable patients were required to provide an 80% power to reject P0 when the true ORR was 15%. With a 10% follow‐up loss rate, a total of 49 patients were required.

PFS was defined as the time from the start date of poziotinib to progression or death from any cause, and OS was defined as the interval from the start date of poziotinib therapy to death from any cause. PFS and OS were evaluated using the Kaplan–Meier curve and were compared using log‐rank test. All statistical analyses were performed using SPSS version 25.0.^17^

## 3 RESULTS

### 3.1 Patient characteristics

From July 2014 to March 2020, a total of 49 patients were enrolled. Patient demographics are listed in Table 1. The median age was 62 years (age range, 21–78 years). The number of male patients was 36 (73%). Patients who never smoked accounted for 41% cases, and among smokers, those with a smoking history of more than 10 years accounted for 53%.

**TABLE 1:** Baseline characteristics (*N* = 49)

Characteristic	Patients, n (%)
Sex	
M	36 (73.5)
F	13 (2.5)
Age, y	
Median(range)	62 (21–78)
Smoking history	
Never smoker	20 (40.8)
Smoker, pack‐years	
≤10	3 (6.1)
>10	26 (53.1)
Primary site	
Oral cavity	14 (28.6)
Oropharynx	14 (28.6)
Hypopharynx	7 (14.3)
Larynx	8 (16.3)
Ethmoid sinus	2 (4.1)
Maxillary sinus	4 (8.2)
Locoregional	16 (32.7%)
Distant	13 (26.5%)
Both	20 (40.8%)
1	11(22.4%)
2	15 (30.6%)
≥3	23 (46.9%)
0	3 (6.1)
1	17 (34.7)
≥2	29 (59.2)
Prior treatment	
None	0 (0.0)
Chemotherapy alone	3 (6.1)
Radiation alone	1 (2.0)
Surgery alone	2 (4.1)
Surgery +RT	0 (0.0)
Surgery +CT	3 (6.1)
Radiation +CT	8 (16.3)
Surgery +RT + CT	32 (65.3)

The most common sites of the primary lesion were the oral cavity (28.6%) and oropharynx (28.6%). Furthermore, 41% of patients had both loco‐regional and distant disease in at least three organs. Three patients (6%) received poziotinib as the first‐line treatment because of borderline impairment of renal function. About two‐thirds (65%) of patients underwent all treatment modalities including surgery, chemotherapy, and radiotherapy before enrolment.

### 3.2 Efficacy and treatment delivery

**FIGURE 1:**
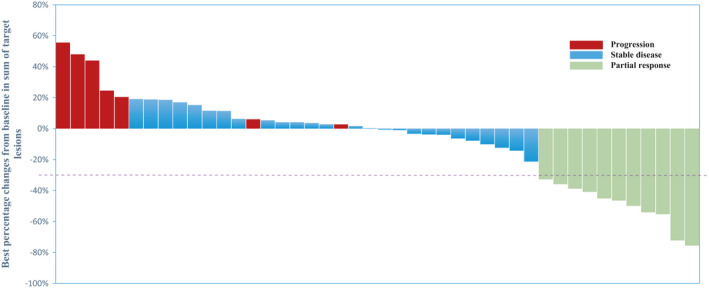
Best response for target lesions by patient, based on maximal percentage changes from the baseline (*N* = 44). Of 49 patients, 5 were excluded from this analysis because follow‐up imaging was not available

The response of 49 patients was evaluated (Table 2). Five patients were not evaluated because of early withdrawal. In addition, 22.4% patients (11/49) showed partial response (PR), 53.1% (26/49) showed stable disease (SD), and 14.3% (7/49) had progressive disease (PD) as the best response (Figure 1). The median duration of treatment was 23.1 weeks (95% CI, 13.5–32.7 weeks). The reasons for treatment discontinuation were disease progression (n = 34, 69%), patient withdrawal (n = 7, 14%), unacceptable toxicity (n = 3, 6%), and death (n = 5, 10%).

**TABLE 2:** Best response by treatment (*N* = 49)

Characteristic	Patients, n (%)
Best response	
Complete response	0 (0.0)
Partial response	11 (22.4)
Stable disease	26 (53.1)
Progressive disease	7 (14.3)
Not evaluated^a^	5 (10.2)
Best overall response rate	
95% CI	22.4% (13.0–35.9)

Abbreviation: CI, confidence interval.

^a^ Response was not evaluable in five patients because of withdrawal from the study.

**FIGURE 2:**
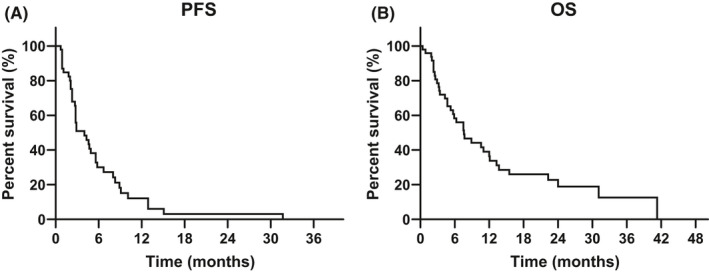
Kaplan–Meier curves of PFS (A) and OS (B) (*N* = 48). PFS, progression‐free survival; OS, overall survival

With a median follow‐up of 7.6 months (95% CI, 8.1–14.3 months), the median PFS was 4.0 months (95% CI, 1.8–6.2 months) and the median OS was 7.6 months (95% CI, 4.4–10.8 months) (Figure 2). Median duration of response was 8.3 months (95% CI 8.1–12.2 months). Among previous chemotherapy regimens, platinum‐based chemotherapy accounted for 72% cases. Patients had also received atezolizumab, durvalumab, M7824, nivolumab, and tremelimumab. Two patients had received PI3K inhibitors (buparlisib and alpelisib) in other clinical trials. Previous chemotherapy regimens administered are described in Supplementary Table 1.

### 3.3 Safety

As for treatment‐related AEs, 48 patients were assessed (Table 3). AEs were mainly grade 1–2 and easily manageable. The most common AEs were acneiform rash (85%) and mucositis (77%). Grade 3 AEs occurred in five patients. Grade 3 acneiform rash occurred in two patients, grade 3 fatigue in one patient, grade 3 mucositis in one patient, and grade 3 lung infection in one patient.

**TABLE 3:** Treatment‐related AEs (*N* = 48)

Toxicity	All grades patients, n(%)	Grade1, 2 patients, n(%)	Grade 3,4 patients, n(%)
Rash acneiform	41 (85%)	39 (82%)	2 (3%)
Paronychia	14 (29%)	14 (29%)	0
Pruritus	12 (25%)	12 (25%)	0
Palmar‐plantar Erythrodysesthesia	4 (8%)	4 (8%)	0
Fatigue	12 (25%)	11 (23%)	1 (2%)
General weakness	2 (3%)	2 (3%)	0
Dry skin	3 (6%)	3 (6%)	0
Conjunctivitis	2 (3%)	2 (3%)	0
Fever	1 (2%)	1 (2%)	0
Lung infection	1 (2%)	0	1 (2%)
Nausea	4 (8%)	4 (8%)	0
Vomiting	1 (2%)	1 (2%)	0
Weight loss	3 (6%)	3 (6%)	0
Paresthesia	1 (2%)	1 (2%)	0
Insomnia	1 (2%)	1 (2%)	0
Mucositis	37 (77%)	36 (75%)	1 (2%)
Diarrhea	27 (56%)	27 (56%)	0
Gastrointestinal pain	1 (2%)	1 (2%)	0
Creatinine increased	2 (3%)	2 (3%)	0
Hyperkalemia	1 (2%)	1 (2%)	0

^a^ One patient was not evaluable because of early withdrawal from the study.

In total, 22 (45.8%) and 16 (33.3%) patients underwent dose reduction to 8 and 6 mg due to AEs, respectively. Common AEs responsible for dose reduction were acneiform rash and mucositis. Four (8.3%) patients discontinued poziotinib therapy due to toxicity, two patients due to skin rash, and others due to lung infection.

### 3.4 Detection of somatic aberrations

**FIGURE 3:**
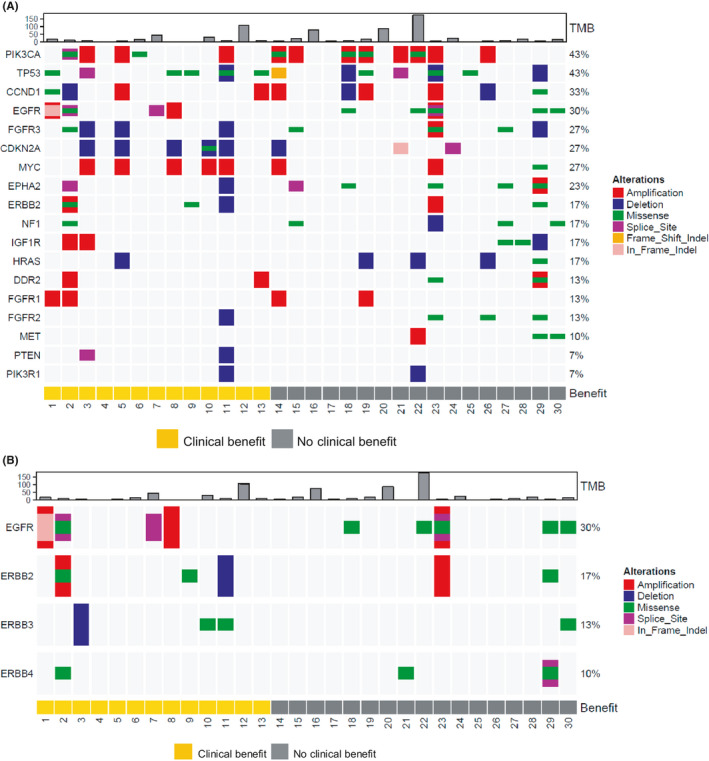
(A) Somatic mutation, gene copy number, and gene expression profiles between the non‐clinically benefitted (PFS <6 months) and clinically benefitted (PFS ≥6 months) (*N* = 30) groups. (B) Somatic mutations and copy number for *EGFR*, *ERBB2*, *ERBB3*, and *ERBB4* (*N *= 30)

Biomarker analyses were available for 30 patients (Figure 3A). Baseline biopsy was not mandatory in this trial, and 14 patients had no archival tissues for biomarker analysis. Target capture sequencing identified a total of 820 point mutations, 6 insertions, and 17 deletions, but in this report, we presented the alterations of the 18 genes previously implicated in the TCGA HNSCC database.^12^ The median sequencing depth in the target regions was greater than 1,000X. The frequency of somatic mutations is illustrated in Figure 3. *TP53* (*R282W*, *R273C*, *R248G*, *G245V*, *Y205C*, *H193R*, *R175H*, *E11Q*) and *PIK3CA* (*N345I*, *R832L*) were the most frequently altered genes (43%) followed by *CCND1* (E70*) (33%) and *EGFR* (*P518L*, *R574L*, *P753Q*, *R836S*, *P848Q*, *G901W*) (30%). Alterations in *ERBB2* (*R978S*), *ERBB3* (*G623E*, *R1173W*), and *ERBB4* (*L713W*, *R393L*, *W10L*), which are HER family genes, were observed in 17%, 13%, and 10% patients, respectively (Figure 3B). Detailed mutation profiles are summarized in Table S3.

Most DNA copy number amplifications and deletions were also observed in genes involved in cell cycle (*CDKN2A* and *CCND1*), receptor tyrosine kinase (*FGFR1*, *EGFR*, and *ERBB2*), and genes related to proliferation (*PIK3CA*). Copy number amplifications were observed in *PIK3CA*, *EGFR*, *ERBB2*, *MYC*, *DDR2*, and *FGFR1*, whereas copy number deletions were observed in *TP53*, *FGFR3*, *CDKN2A*, *HRAS*, *FGFR2*, *PTEN*, and *PIK3R1* (Figure 3A,B).

Additional clinical information on tissues used for target capture sequencing is provided in Table S2.

### 3.5 Association of somatic alterations with clinical outcomes

**FIGURE 4:**
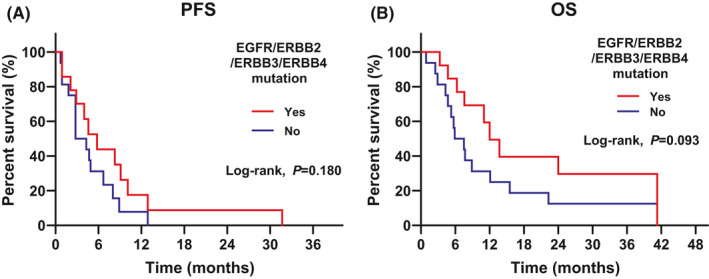
Kaplan–Meier curves of PFS (A) and OS (B) according to the *EGFR*/*ERBB2*/*ERBB3*/*ERBB4* mutation status (*N *= 30). PFS, progression‐free survival; OS, overall survival

Kaplan–Meier curves of median PFS (4.0 months) and OS (7.6 months) are shown in Figure 1. There was no difference in the frequency of somatic mutations in *EGFR*, *ERBB2*, *ERBB3*, and *ERBB4* between the clinically benefitted and non‐benefitted groups. The median PFS (5.8 vs. 2.8 months; log‐rank test, *p* = 0.180; Figure 4A) and median OS (12.0 vs. 5.9 months; log‐rank test, *p* = 0.093; Figure 4B) were not different according to the presence of HER family gene mutations. Regarding other genetic alterations in the PIK3CA/Akt pathway, cell cycle machinery, and FGFR pathway, there was no statistical difference in survival according to the presence of alterations.

## 4 DISCUSSION

We investigated the efficacy and safety of poziotinib in heavily treated R/M‐HNSCC and identified translational biomarkers related to clinical response to poziotinib. Characterization of somatic mutation, DNA copy number, and gene expression was performed.

Patients with HNSCC exhibiting disease progression after platinum‐based chemotherapy have poor prognosis and limited treatment options.^8^ Based on the results of the EXTREME study that showed overall survival benefits with no decrease in the quality of life, the research interest on EGFR inhibitors has increased.^7^

However, despite numerous research efforts targeting EGFR, drugs developed to date have shown limited activity in patients with HNSCC. Suppression of EGFR with tyrosine kinase inhibitors including gefitinib, erlotinib, and lapatinib showed a limited response.^18^, ^19^, ^20^, ^21^ As a single agent, cetuximab in R/M‐HNSCC showed limited activity (ORR13%, time to progression of 70 days; ref.12).^7^ The drug with higher ORR was followed by cetuximab and 500 mg of gefitinib. However, 500mg of gefitinib in R/M‐HNSCC also reached only 10.6% ORR, 3.4 months PFS, and 8.1 months OS.^22^ In Phase 2 study with lapatinib, a competitive reversible inhibitor of EGFR and ERBB2, no complete or partial responses were observed, and stable disease was the best response.^23^

Poziotinib is considered a new treatment option as a promising TKI in carcinomas with *EGFR* mutations.^24^ Compared with previous studies on EGFR TKIs, our study showed comparable data with the ORR of 22.4%, median PFS of 4.0 months, and OS of 7.6 months. Among patients with R/M‐HNSCC who exhibited disease progression after platinum‐based chemotherapy or who were not eligible for platinum‐based chemotherapy, treatment with poziotinib showed longer survival than that with the standard therapy.

Poziotinib showed similar toxicity to other pan‐HER inhibitors, but it might be related to early dose reduction due to AEs. The most common AEs were acneiform rash and mucositis, and most patients showed manageable toxicity of grades 1–2. These AEs could be managed through supportive care and oral medications.

Our study did not show an association between any *EGFR* mutation and the response to poziotinib. This may be because of the small sample size of our study. Although there was no statistical difference between patients with or without *EGFR* mutation, median OS increased in patients with *EGFR* mutation.

Recently, numerous studies on immunotherapy in patients with R/M‐HNSCC showed noticeable responses.^8^, ^25^ The recurrence and metastasis of HNSCC are facilitated by immune evasion,^26^ which is mediated in part by the expression of programmed death‐ligand (PD‐L1 and PD‐L2), which binds to the T‐cell suppressive immune checkpoint receptor PD‐1.^22^, ^27^

In a preclinical study, activation of the EGFR pathway induced PD‐L1 expression and enhanced susceptibility of the lung tumors to PD‐1 blocker, suggesting that a combination of PD‐1 blocker with EGFR TKIs may be a promising treatment to extend the duration of response and delay resistance.^28^, ^29^ Thus, the combination of EGFR TKI with immunotherapy may show better responses in HNSCC. In addition, in case of elderly patients or patients who are ineligible for cytotoxic chemotherapy, the combination of immunotherapy and poziotinib is expected to be effective without severe toxicity. However, these attempts (NCT03695510) should be further validated in prospective trials.

The limitation of this study is that it is a single‐center, single‐arm study with a relatively small number of patients available for response assessment. The response was not evaluated in five patients because of early withdrawal. One patient withdrew consent without taking poziotinib. Two patients withdrew consents before the first response evaluation due to decreased general condition. Another patient discontinued at the discretion of the investigator due to lung infection of grade 3 or higher and general weakness. The last patient died of sudden cardiac death, which was not considered to be related to the drug. Additionally, frequent low‐grade and serious grade 3 or greater skin rash and mucositis need careful monitoring and frequent intervention. However, we believe that pre‐emptive dose reduction and active prevention and education of adverse events could raise the adherence and efficacy of this drug.

In conclusion, compared with other previous EGFR TKIs, poziotinib showed clinically meaningful efficacy with manageable toxicity in patients with platinum‐refractory R/M‐HNSCC. Owing to the small number of tissues available for targeted capture sequencing, we could not identify useful biomarkers involved in the response to poziotinib. The identification of molecular markers that could predict clinical response to targeted therapy will aid in the development of personalized targeted treatment, which should be the focus of future studies.

## CONFLICT OF INTEREST

The authors have no conflicts of interest to disclose.

## AUTHOR CONTRIBUTIONS

**Ji Hyun Lee**: contribution to the data acquisition, responsibility for writing the paper, and statistical analysis. **Seong Gu Heo:** software, statistical analysis, **Beung‐chul Ahn:** participation in patient management and data collection. **Min hee Hong:** participation in patient management and data collection, **Byoung Chul Cho:** conceptualization, participation in patient management and data collection, **Sun Min Lim:** conceptualization, study design, participation in patient management and data collection, responsibility for writing the paper. **Hye Ryun Kim**: conceptualization, study design, participation in patient management and data collection, responsibility for writing the paper. All authors reviewed the paper and approved the final version.

## ETHICAL APPROVAL STATEMENT

This study was approved by the Institutional Review Board of Severance Hospital (4–2013–0794). The study conforms to the principles for research outlined in the Declaration of Helsinki.

## Data Availability

The data that support the findings of this study are available from the corresponding author upon reasonable request.
